# Internal-Fixation Hardware Infection With Corynebacterium jeikeium

**DOI:** 10.7759/cureus.17676

**Published:** 2021-09-03

**Authors:** Lintu Ramachandran, Moamen Al Zoubi, Ayobami Olaleye

**Affiliations:** 1 Internal Medicine, Javon Bea Hospital, Rockford, USA; 2 Infectious Diseases, MercyHealth Hospital, Rockford, USA

**Keywords:** corynebacterium jeikeium, hardware infection, c jeikeium, internal-fixation hardware infection, literature review of disease

## Abstract

*Corynebacterium* is a rare cause of prosthetic joint infections (PJIs) and infection after fracture fixation (IAFF). We present a case of a patient who developed *Corynebacterium jeikeium-*associated IAFF three weeks after his fracture fixation. Due to its slow-growing nature, surgical cultures remained negative after 72 hours and grew only on day 5. We highlight that physicians should have *Corynebacterium*-associated infection in their differential in such cases, especially when the cultures remain negative after 72 hours. We also review the literature of PJI and implant-associated infection with *C. jeikeium *and discuss the antibiotic resistance patterns and some microbiological considerations associated with *C. jeikeium.*

## Introduction

Corynebacteria are gram positive microorganisms that are present in normal human skin flora. Although an uncommon cause of pathogenesis, case reports have linked *Corynebacterium* to endocarditis, sepsis, and even osteomyelitis [1]. *Corynebacterium* species commonly isolated in the clinical laboratory include* C. amycolatum*, *C. aurimucosum*, *C. glucuronolyticum*, *C. jeikeium*, *C. pseudodiphtheriticum*, *C. striatum*, *C. tuberculostearicum*, and *C. urealyticum*. *C. glucuronolyticum* and* C. urealyticum* are involved predominantly in urinary tract infection, while *C. pseudodiphtheriticum *is implicated in respiratory tract infections [2]. The remaining five commensal flora species are potential causes of device-associated infections, including prosthetic joint infections (PJIs) [3]. Corynebacteria that cause orthopedic device-associated infections are not usually identified to the species level, and specific *Corynebacterium* species involved in PJIs and infection after fracture fixation (IAFF) are not definitively known. In cases of *C. jeikeium-*associated PJI and IAFF, often diagnosis and appropriate treatment can be delayed due to slow growth of *C. jeikeium* and the antibiotic resistance associated with *C. jeikeium.* We present the case of a patient who underwent intramedullary nail placement for a periprosthetic fracture and subsequently developed *C. jeikeium* prosthetic infection. The patient was treated with debridement and partial hardware removal followed by six weeks of intravenous (IV) vancomycin. 

## Case presentation

Our patient was a 52-year-old male with a past medical history of cerebral palsy, essential hypertension, depression, and knee arthritis status post total knee arthroplasty, who presented to the emergency department after a mechanical fall. He was found to have a left distal femoral shaft fracture above the total knee arthroplasty which was fixed with an intramedullary rod placement. The patient was discharged home in a stable condition. Three weeks later, he developed increased redness and swelling at the surgical site. Vital signs were all stable on admission. Physical examination was significant for a 10 cm post-surgical wound around the left knee with mild dehiscence. Granulation tissue with drainage of serosanguineous fluid was noted along with mild erythema around the surgical site. Laboratory results including white blood cell count, erythrocyte sedimentation rate, and C-reactive protein were all normal at 3,900/µL, 1 mm/h, and 2 mg/L, respectively. Blood cultures were obtained and remained negative at 72 hours. He was taken to the operating room and underwent extensive excisional debridement including skin, muscle, and bone. There was one screw of the nail that was loose and, therefore, removed. Antibiotic (gentamycin)-impregnated cement beads were inserted. Gram stain from intraoperative culture showed no organisms. He was started empirically on cefepime 2 g IV every 8 hours and vancomycin 1 g every 12 hours postoperatively. After four days, there was no growth from the surgical cultures. Microbiology lab was asked to hold the culture for 10 days for the concern of slow-growing organism. The patient was discharged with ceftriaxone 2 g every 24 hours through a PICC line. Five days after discharge, aerobic cultures grew *Corynebacterium* species. The specimen was sent to Mayo Clinic (Rochester, Minnesota) for further testing. Quantitative antibiotic susceptibility testing was performed to determine the isolate’s minimum inhibitory concentration (MIC) to various drugs. The *Corynebacterium* species was identified as *C. jeikeium*, which was resistant to penicillin, ceftriaxone, and doxycycline but susceptible to vancomycin. The susceptibilities and the MICs were as listed in Table 1. 

**Table 1:** Antibiotic Susceptibilities and MICs MIC, minimum inhibitory concentration.

Organism: *Corynebacterium jeikeium*		
Antibiotic	MIC (µg/mL)	Interpretation
Penicillin	>8	Resistant
Ceftriaxone	>2	Resistant
Meropenem	>8	Resistant
Vancomycin	≤1	Susceptible

The patient’s antibiotic was switched to vancomycin IV 1.5 g every 12 hours with a target trough level of 15-20 µg/mL. His treatment course remained uneventful, and he completed a six-week course of vancomycin. The patient did not follow up with the orthopedic surgeon or the infectious disease team due to fear of COVID-19. One year later, the patient presented to the emergency department with draining wound and significant fluctuance above his left distal femoral fracture. He was found to be septic with *Staphylococcus aureus* bacteremia. He underwent irrigation and excisional debridement of his wound along with antibiotic (gentamycin) bead exchange. However, due to persistent sepsis, the patient required above-knee amputation to control his infection. His operating room cultures grew *S. aureus* as well. The patient completed two weeks of IV cefazolin postoperatively.

## Discussion

The exact incidence of *Corynebacterium*-associated PJI is unclear. A prospective study conducted by Mayo Clinic reviewed operating room cultures between 2006 and 2011 and found a total of five *Corynebacterium *spp PJI out of 354 patients. None of the five patients specifically had *C. jeikeium* [4]. A literature review conducted in 2005 found seven total cases of *C. jeikeium-*associated PJI [5]. A subsequent study published in 2012 used 19 known *Corynebacterium *spp isolates, collected between 1999 and 2008, to create a novel detection assay. Two of the 19 isolates were *C. jeikeium* [6]. Our literature search did not show any reported cases of for *C. jeikeium* PJI in humans since the 2012 study. The only other reported case, since 2012, is that of synovial fluid infection with *C. jeikeium* reported in a 45-day-old calf [7]. *Corynebacterium*-associated IAFF is also relatively rare, especially with *C. jeikeium*. Our literature search only showed one reported case of *C. jeikeium* nailed tibial fracture [8]. Our case is the second reported case of *C. jeikeium* IAFF.

The treatment strategy in PJI is based on the stability of the prosthesis, timing of the infection, and the extent of the infection. The different types of intervention include surgical debridement with retention of the prosthesis, one-stage implant exchange, two-stage exchange with a spacer and a short course of antibiotics, two-stage exchange without a spacer and a long course of antibiotics, and suppressive long-term therapy with antibiotics and without surgical intervention in some cases [9]. While most of the treatment strategies for AIFF are derived from PJI treatment plans, there are some major differences.

 In PJI, the treatment goal is eradication of infection and a sterile implant. In IAFF, treatment goals include fracture consolidation and prevention of chronic osteomyelitis. Stable hardware can be maintained in a patient without uncontrolled sepsis who has no signs of chronic osteomyelitis. In the case of early infection or acute hematogenous infection after an uneventful postoperative period, rapid diagnosis and immediate surgical and antimicrobial treatment improve the outcome. Before surgery, the following factors must be assessed: viability of the soft tissue and bone, bone stability, fracture healing, implant stability, duration of the infection, and magnitude of the soft tissue defect [10]. Generally, stability of bone fragments is required for union. Possible treatment options include debridement with implant retention along with 12 weeks of antibiotic treatment, one-stage exchange with 12 weeks of antibiotics, two-step exchange with six weeks of antibiotic treatment after each step, and debridement alone with long-term antibiotics until removal of implant [11]. In our patient’s case the intramedullary nail was left intact and only a loose screw was removed. He was successfully treated with the screw removal and IV vancomycin alone.

*C. jeikeium* has been shown to have multidrug antibiotic resistances including resistance to β-lactamases, macrolides, aminoglycosides, and fluoroquinolones [12]. The presence of two ampicillin-insensitive cross-linking enzymes has been shown to be the mechanism of action behind the β-lactamase resistance of *C. jeikeium* [13]. Modification of the genes that code for the 50S ribosomal subunit has led to resistances against macrolide, lincosamide, and streptogramin B in *C. jeikeium* [14]. Fluoroquinolones act by binding to the DNA gyrase of the bacterium, thereby preventing bacterial transcription and further replication. Novel modifications in the quinolone-resistance-determining region near the *gyrA* gene, which encodes for the DNA gyrase, have been documented as the reasoning behind *Corynebacterium*’s fluoroquinolone resistance [15]. Antibiotic resistance of *C. jeikeium* to meropenem has also been documented in the literature [16]. Our patient’s surgical cultures demonstrated resistances to penicillin, ceftriaxone, and meropenem with sensitivity to vancomycin only. He clinically improved after the initiation of vancomycin, but he eventually required an above-knee amputation secondary to Staphylococcal infection.

Lipophilic Corynebacteria, such as *C. jeikeium*, are fastidious and slow growing [17]. In our case, the patient did not have positive cultures until after five days. A plausible explanation for the slow growth of *C. jeikeium* could be the bacteria’s ability to enter dormant state. Studies have demonstrated the in vitro ability of *C. jeikeium* to remain dormant and in a non- culturable state. In such a state, the bacterium retains the ability to proliferate, but does not produce colonies on agar plate, thereby being unable to detect on standardized cultures [18].

The fact that our patient had IAFF after three weeks after surgery makes our case unique, especially considering the slow-growing nature of *C. jeikeium.* Clinicians should have a high index of suspicion for *Corynebacterium*-associated infection when surgical cultures remain negative after 4-5 days. While there are no differences in clinical practice in the antibiotic treatment between *Corynebacterium*-associated PJI and IAFF, there are no direct comparison studies between the two for determining the length of the treatment. Further research, more specifically, direct head-to-head studies comparing the treatment duration for IAFF and PJI in *C. jeikeium,* would most certainly be beneficial.

## Conclusions

In conclusion, we present a rare case of *C. jeikeium* IAFF. Due to the inherent resistance of *C. jeikeium* to several classes of antibiotics, the drug of choice for treatment remains vancomycin currently. The slow-growing nature of *C. jeikeium* and its ability to remain dormant make *C. jeikeium* infections difficult to diagnose and potentially fatal. Clinicians should have a high index of suspicion for *Corynebacterium-*associated infection in patients when their operating room cultures remain negative even after 4-5 days.
